# Novel preclinical models of topical PrEP pharmacodynamics provide rationale for combination of drugs with complementary properties

**DOI:** 10.1186/1742-4690-10-113

**Published:** 2013-10-24

**Authors:** Pedro MM Mesquita, Priya Srinivasan, Todd J Johnson, Rachna Rastogi, Tammy Evans-Strickfaden, Michael S Kay, Karen W Buckheit, Robert W Buckheit Jr, James M Smith, Patrick F Kiser, Betsy C Herold

**Affiliations:** 1 Departments of Pediatrics and Microbiology &Immunology, Albert Einstein College of Medicine, Bronx, NY, USA; 2 Laboratory Branch, Division of HIV/AIDS Prevention, National Center for HIV/AIDS, Viral Hepatitis, STD, and TB Prevention, Centers for Disease Control and Prevention, Atlanta, GA, USA; 3 Department of Bioengineering, University of Utah, Salt Lake City, UT, USA; 4 Department of Biochemistry, University of Utah, Salt Lake City, UT, USA; 5 ImQuest BioSciences Inc, Frederick, MD, USA

**Keywords:** HIV, Pre-Exposure Prophylaxis, Intravaginal ring, Microbicide, Preclinical models

## Abstract

**Background:**

The limited success of recent HIV topical pre-exposure prophylaxis clinical trials highlights the need for more predictive models of drug efficacy that better simulate what may happen during sexual exposure. To address this gap, we developed complementary *in vitro* models to evaluate the ability of drugs to retain anti-HIV activity if cells were washed with seminal plasma (simulating what may happen following exposure to ejaculate), and to protect drug-naive T cells (representing newly recruited immune cells) co-cultured with explants that had been pretreated with drug. We focused on tenofovir disoproxil fumarate (TDF), the non-nucleoside reverse transcriptase inhibitors dapivirine (DPV) and IQP-0528, and the entry inhibitors maraviroc (MVC) and the D-peptide chol-PIE-12 trimer (PIE12). Studies were extended to macaques and the ability of cervical biopsies obtained from animals treated with an intravaginal ring formulation of IQP-0528 to protect *ex vivo* co-cultured T cells was determined. The antiviral activity of cervicovaginal lavage samples against a primary Clade C isolate was also measured and correlated with drug levels.

**Results:**

Cells exposed to TDF were equally protected from HIV whether or not the drug-treated cells were washed with medium or seminal plasma prior to challenge. In contrast, several-fold higher concentrations of NNRTIs and entry inhibitors were needed to attain similar levels of HIV inhibition following a wash with seminal plasma. Conversely, the NNRTIs and PIE12, but not TDF or MVC, were effectively transferred from *ex vivo* treated explants and protected co-cultured T cells. Biopsies obtained from IQP-0528 ring-treated macaques also protected co-cultured T cells with viral inhibition ranging from 42-72%. Antiviral activity correlated with the concentration of drug recovered. Combinations of TDF with IQP-0528 protected in both *in vitro* models.

**Conclusions:**

Together, these models suggest that intracellularly retained drugs such as TDF may protect resident immune cells following coitus but sustained delivery may be required to protect immune cells subsequently recruited into the genital tract. Sustained delivery may also be critical for NNRTIs, which are rapidly transported out of cells and could be lost following sexual intercourse. An ideal approach may be a combination of drugs with complementary bioavailability profiles formulated for sustained delivery.

## Background

The development and implementation of strategies to prevent HIV and other sexually transmitted infections (STI) in high-risk populations are a public health imperative. There have been several successes in the battle to develop safe and effective prevention modalities, including oral or topical pre-exposure prophylaxis (PrEP), as highlighted by the recent FDA approval of oral Truvada, a fixed-dose combination of two reverse transcriptase inhibitors (RTIs): tenofovir disoproxil fumarate (TDF) and emtricitabine. Orally administered Truvada or TDF provided significant, albeit variable, protection in four separate clinical trials [1-4], but was ineffective in two others [5,6], likely reflecting poor adherence by study participants [7]. Encouraging results were also obtained in a clinical trial with 1% tenofovir (TFV) gel applied vaginally before and after coitus [8]. However, in a subsequent trial, daily application of the same TFV gel formulation was not protective; the disappointing outcome was attributed, at least in part, to poor adherence [9]. Together, these studies illustrate that it is clearly possible to prevent sexual transmission with topical application of antiretrovirals (ARVs) but low adherence to the gel treatment regimens may impede the ability to measure efficacy. Thus, the challenge in establishing a clinically efficacious HIV prevention method may be largely rooted in both human behavior and biology.

The formulation of PrEP has the potential to address both the behavioral and biological determinants of HIV prevention. Sustained delivery systems, for example, may promote adherence, but efficacy may also be impacted by the ability of the formulation to deliver and maintain sufficient concentration of drug at the sites of infection. For example, while bioactive drug was detected in cerviocovaginal lavage (CVL) samples obtained from women who applied 0.5% PRO 2000 gel in the absence of sex, there was a significant reduction in the concentration of drug recovered and the anti-HIV activity of CVL in samples obtained shortly after coitus [10]. We speculate that the observed reduction in anti-HIV activity of postcoital CVL samples reflected drug loss due to leakage, redistribution and dilution with ejaculate. At the molecular level, activity was likely reduced due to interactions of the drug with molecules present in semen that altered PRO 2000’s ability to bind to viral glycoproteins [11] and thus contributed to the failure of the drug to protect against HIV (or HSV-2) acquisition [12].

In the current study, we sought to determine the *in vitro* efficacy of a number of PrEP ARVs using complementary models designed to evaluate the pharmacokinetics (PK) and pharmacodynamics (PD) of drugs. The models focused on the potential washout effects of an ejaculate and the need to protect both resident and immune cells recruited into the genital tract. The importance of newly recruited immune cells is illustrated by the observation that there is an increase in HIV target cells including monocyte/macrophages and dendritic cells in cervical biopsies following unprotected sexual intercourse compared to samples obtained from abstinent women [13]. We tested these *in vitro* models using drugs being advanced as topical PrEP candidates that differ in site (cell surface or intracellular) and mechanism (reverse transcriptase inhibitors (RTIs) and entry inhibitors), as well as need for intracellular modifications (e.g., phosphorylation of TFV). The drugs included TDF, the more potent prodrug of TFV that exhibits greater cellular uptake than TFV and has recently been successfully formulated as an IVR and shown complete protection in a repeat low dose macaque challenge model [14,15]; the non-nucleoside RTIs (NNRTIs), dapivirine (DPV) and IQP-0528, which have both been formulated as gels and IVRs [16-19] and, unlike TDF/TFV, do not require intracellular modification; and two entry inhibitors: maraviroc (MVC), a CCR5 coreceptor antagonist, which is being evaluated in clinical trials as an IVR alone and in combination with DPV, and a cholesterol-modified D-peptide that targets the HIV gp41 N-trimer pocket, chol-PIE-12 trimer (PIE12), which is in formulation development [20].

Presumably, entry inhibitors must be present at sufficiently high concentrations at the cell surface of immune targets in the genital tract following intercourse, whereas RTIs must be retained intracellularly within HIV target cells following coitus. Both types of drugs must be accessible to resident immune cells and to cells recruited into the genital tract in response to chemokines and other inflammatory signals released in response to sex or other environmental stimuli. To model these clinical scenarios *in vitro*, the ability of drugs to retain anti-HIV activity if cells were washed with seminal plasma (SP) (simulating a washout following exposure to ejaculate) and to protect drug-naive T cells (representing newly recruited immune cells) co-cultured with explant tissue that had been pretreated with drug, were evaluated. We then extended the studies to *in vivo* models and tested the PK/PD relationships in CVL and cervical biopsies obtained from macaques following 14-day treatment with an IVR delivering IQP-0528 [16]. Taken together, the results of the studies indicate that combinations of drugs with complementary PK/PD properties and formulated for sustained drug delivery may provide the greatest protection against sexual transmission of HIV.

## Results and discussion

### Differential intracellular drug retention

**Figure 1:**
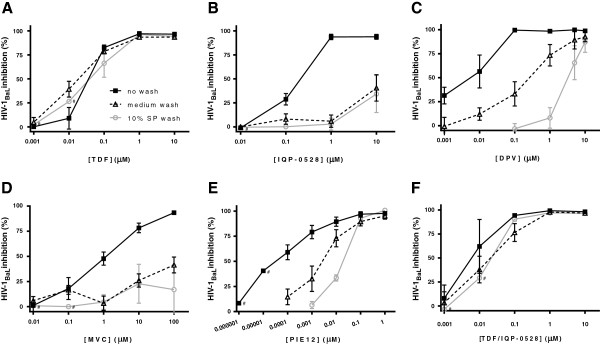
**Differential intracellular drug retention.** JT-CCR5 cells were exposed to drug (**A**- TDF; **B**- IQP-0528; **C**- DPV; **D**- MVC; **E**- PIE12; **F**- TDF + IQP-0528) in complete medium for 24 h prior to challenge with approximately 10^3^ TCID_50_ HIV-1_Ba-L_. Virus challenge was performed in the presence of drug (black lines, closed squares), after washing with serum-free medium (hatched black lines, open triangles) or after washing with serum-free medium containing 10% seminal plasma (SP) (grey lines, open circles). HIV replication was assessed by measuring p24 antigen in culture supernatants 6 days post-infection. Data are presented as percent inhibition relative to cells challenged in the absence of treatment and are means ± SEM obtained from at least three independent experiments where each condition was tested in triplicate, except where noted # (one experiment).

To model the potential effects that an ejaculate may have on drug activity *in vitro,* we exposed Jurkat-Tat-CCR5 T cells (JT-CCR5) to varying concentrations of TDF, IQP-0528, DPV, MVC and PIE12 for 24 h and then either washed the cells with medium containing 10% SP, medium alone or no wash (control) prior to challenge with HIV-1_Ba-L_. The highest concentrations of drugs tested had no impact on cell viability assessed by measuring the metabolism of 3-(4,5-dimethylthiazol-2-yl)-5-(3-carboxymethoxyphenyl)-2-(4-sulfophenyl)-2H-tetrazolium (MTS) (data not shown). Consistent with prior studies when HIV was added to cultures in the absence or presence of SP [21], TDF (at concentrations greater than or equal to 0.1 μM) retained its antiviral activity and was not impacted by washing with either medium or SP (Figure 1a). This likely reflects the rapid uptake and intracellular metabolism of the drug to TFV-diphosphate (TFV-DP), which has a long intracellular half-life [22].

In contrast, there was a significant reduction in inhibitory activity for the NNRTIs and entry inhibitors, as illustrated by a shift to the right in the dose response curves if the cells were washed with either medium or SP prior to viral challenge (Figure 1b-e). At the highest concentrations tested (10 and 100 μM, neither IQP-0528 or MVC completely protected the cells from infection, although it is possible that higher concentrations might overcome the washout effect. The shift in the dose response curve for the NNRTIs is consistent with the dynamic movement of drugs into and out of cells in response to intracellular and extracellular concentration gradients [23]. Notably, DPV was affected more by a wash with 10% SP compared to medium, suggesting that the drug may bind to seminal proteins, as has been reported for serum proteins [24]. For example, 1 μM DPV failed to inhibit HIV infection if the cells were washed with SP prior to HIV challenge, whereas the same concentration of drug provided 73.9 ± 13.8% (mean ± SEM) protection if the cells were washed with medium alone (Figure 1c). Importantly, the combination of equal concentrations of TDF and IQP-0528 resulted in HIV inhibition comparable to what was observed following exposure to TDF alone. This suggests that the presence of a NNRTI does not impact TDF uptake and intracellular retention (Figure 1f).

The CCR5 co-receptor antagonist MVC, which has previously been shown to be active in the low nanomolar range if drug is present throughout the experiment [25], was only active in the low μM range under the current experimental conditions (cells washed 2 h after viral inoculation) (Table 1). When MVC was re-added to cultures following the washing step, the antiviral activity observed was consistent with published data [25] (not shown). To determine whether the higher concentrations of MVC needed in these studies might reflect the greater expression of CCR5 at the cell surface of JT-CCR5 cells compared to primary cells, parallel experiments were conducted using human PBMCs. Flow cytometry confirmed greater expression of CCR5 on JT-CCR5 cells compared to PBMCs (99% of JT-CCR5 cells expressed CCR5 compared to 30-40% of PHA-activated PBMCs), but there was no difference in the antiviral activity of MVC (data not shown). Moreover, the inhibitory activity of MVC was significantly reduced if the JT-CCR5 cells were washed with medium or medium containing SP prior to infecting with virus (Figure 1d). The finding that higher concentrations of MVC are needed if drug is only present prior to HIV exposure combined with the loss of activity observed following a wash with medium or SP suggest that protection will require sustained MVC delivery.

**Table 1:** Human cervical tissue as a drug reservoir for protection of T cells

**Drug**	**JT-CCR5 Infection**		**Co-culture JT-CCR5 &explant tissue**	
	**IC**_**50**_**(μM)**	**IC**_**90**_**(μM)**	**IC**_**50**_**(μM)**	**IC**_**90**_**(μM)**
TDF	0.04 ± 0.01	0.13 ± 0.02	>10.0	>10.0
IQP-0528	0.19 ± 0.07	0.43 ± 0.16	2.9 ± 0.7	10.1 ± 0.7
DPV	0.01 ± 0.01	0.03 ± 0.02	0.4 ± 0.2	1.1 ± 0.3
MVC	1.98 ± 0.85	54.5 ± 13.8	>100	>100
Chol-PIE12 trimer	<0.0001	0.009 ± 0.007	0.008 ± 0.004	0.09 ± 0.01
TDF + IQP-0528	0.01 ± 0.01	0.03 ± 0.02	0.5 ± 0.2	9.5 ± 0.8

JT-CCR5 cells were challenged with HIV-1_Ba-L_ in the presence of indicated drugs. Alternatively, human ectocervical explants were exposed to the same panel of drugs for 24 h, washed, and then co-cultured with JT-CCR5 cells prior to challenge with virus. IC_50_ and IC_90_ values were calculated from at least two experiments with each condition tested in triplicate and are means ± SEM.

There was also a loss of activity for PIE12 following washing, but complete inhibition of HIV infection was still achieved at concentrations less than 1μM, even after a wash with SP (Figure 1e). The membrane-localizing cholesterol group, which is conjugated to the PIE12-trimer by a PEG linker, may contribute to potent antiviral activity of this entry inhibitor even after wash-out with medium or SP [20].

### Co-culture model

**Figure 2:**
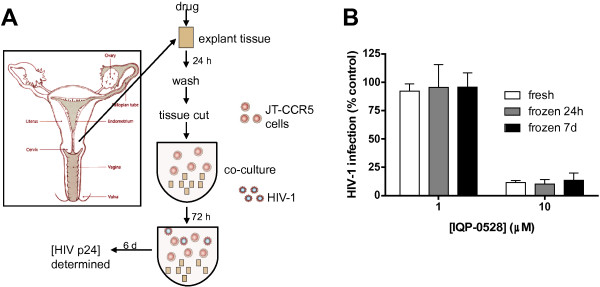
**Co-culture model. (A)** Schematic representation of model. Drug-exposed human explant tissue is washed, minced and co-cultured with HIV-susceptible cells (JT-CCR5 cells) for 72 h to allow drug present in the tissue to be transported into cells prior to challenge with HIV-1_Ba-L_. **(B)** Ectocervical explants were exposed to indicated concentrations of IQP-0528 for 24 hours. Tissue was washed and either immediately co-cultured with JT-CCR5 cells for 72 h prior to challenge or stored at -80C for 24 h or 7 d before co-culture and challenge. HIV replication was assessed by measuring p24 antigen in culture supernatants 7 days post-infection. Data are presented as the percentage infection relative to co-cultures challenged in the absence of drug treatment and are means ± SEM obtained from at least two independent experiments using tissue from different donors where each condition was tested in triplicate.

Cell cultures provide a first approximation of potential drug activity, but do not reflect the complexities found in human genital tract tissue. While direct challenge of tissue would provide information about how well the drug can protect resident target cells from HIV, reproducibility, variability in the number and activation status of immune cells, and the need to work with fresh tissue has limited the feasibility of using direct challenges as a model of PD in clinical trials [26,27]. To circumvent these problems, we developed a model to test how well tissue serves as a reservoir to protect immune cells that might be recruited into the genital tract in response to various environmental stimuli [13]. Human ectocervical explant tissue was treated *ex vivo* with varying concentrations of the panel of drugs described above, washed, and then minced and co-cultured with JT-CCR5 cells prior to being challenged with HIV-1_Ba-L_ (Figure 2a). The ability of tissue-associated drug to protect the co-cultured cells from infection was monitored by p24 ELISA. The concentrations of drugs used during tissue exposure that inhibited 50% (IC_50_) and 90% (IC_90_) of HIV infection in this co-culture model were compared to the concentrations needed to inhibit direct infection of JT-CCR5 cells (Table 1). Cells co-cultured with tissue that had been exposed to TDF were not protected from viral infection, which is consistent with the intracellular trapping of TFV-DP and the data presented in Figure 1. MVC also failed to protect co-cultured T cells.

In contrast, pretreatment of explant tissue *ex vivo* with IQP-0528, DPV or PIE12 provided significant protection against infection of the co-cultured T cells, indicating the potential ability of the drugs to protect newly recruited immune cells. The multilayered nature of the tissue likely provides a sufficiently large reservoir of these drugs, which are released to protect co-cultured T cells but not during the short washing step prior to co-culture. The combination of equal concentrations of TDF and IQP-0528 provided complete protection in both the washout (Figure 1f) and the co-culture (Table 1) model. In the former model, efficacy reflects the resistance of intracellular TFV-DP to a wash, whereas in the latter, the protection reflects the rapid transit of IQP-0528 out of the tissue and into the JT-CCR5 cells. These findings illustrate the advantage of combining drugs with complementary PK/PD properties.

To address the feasibility of applying the co-culture model to clinical studies, we compared co-cultures of tissue frozen and stored at -80°C (for 24 h or 7 days) following drug treatment to co-cultures of fresh tissue. Fresh ectocervical explants were treated with IQP-0528, washed and then either stored at -80°C or immediately processed as described above and co-cultured with JT-CCR5 T cells. IQP-0528 was protective under all of these conditions (Figure 2b).

### PK/PD of IQP-0528 IVR in pig-tailed macaques

Treatment of tissue with drug *ex vivo* may not reflect the tissue levels achieved *in vivo*. Therefore, we applied our models to biological samples obtained from non-human primates (NHPs) that participated in a 14-day study of a 10% (w/w) polyurethane IVR that delivered an average of 200 μg/day of IQP-0528 [16]. Four animals were treated with the active ring and 2 with placebo IVR devices. We examined drug levels (PK) and antiviral activity (PD) in CVL and cervical biopsies (using the co-culture model) obtained proximal and distal to the site of ring placement near the cervix, on Days 7 and 14 post ring insertion. Significantly higher concentrations of drug were detected in Day 14 proximal and distal biopsies compared to Day 7 biopsies (Table 2; Mann Whitney test; p = 0.001), indicating that the NNRTI accumulated in genital tract tissue over time and may provide a local source of drug for recruited cells. In contrast, there was little difference in levels measured in CVL between Day 7 and 14. This is consistent with what we observed in a study comparing different loading doses in which rings loaded with high concentrations of pyrimidinediones displayed near time-independent release rates, likely due to saturation-dependent drug release [16].

**Table 2:** IQP-0528 levels in macaque samples

		**Macaques**				**Mean ± SEM**
		**PMD2**	**PKP1**	**PHC2**	**PTE2**	
CVL	D7	1490	1290	730	1930	1360 ± 249
	D14	1600	510	600	1050	940 ± 250
Cervical tissue	Proximal D7	1.03	1.01	6.16	5.24	3.36 ± 1.36
	Proximal D14	33.32	36.68	12.46	29.87	28.08 ± 5.39
	Distal D7	0.45	0.66	0.79	1.50	0.85 ± 0.23
	Distal D14	28.95	2.38	5.66	21.62	14.65 ± 6.35

IQP-0528 concentration in cervicovaginal lavage (CVL) samples (μg/mL) or in proximal or distal cervical biopsy tissue samples (ng/mg of tissue).

**Figure 3:**
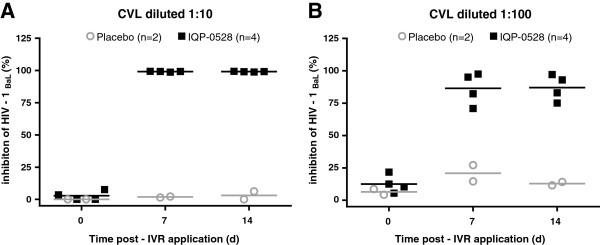
**Antiviral activity in NHP CVL.** TZM-bl cells were challenged with approximately 10^3^ TCID_50_ HIV-1_Ba-L_ in the presence of 1:10 **(A)** or 1:100 **(B)** dilutions of CVL collected from pig-tailed macaques at Day 0 (baseline) and 7 and 14 days post-insertion of IVR. Four animals were exposed to IVRs loaded with 10% (w/w) IQP-0528 (squares) and two animals received placebo IVRs (circles). HIV infection was assessed by measuring luciferase activity 48 h post-challenge. Data are presented as the percent inhibition relative to control cells challenged in the absence of CVL and are means obtained from two independent experiments where each condition was tested in triplicate. The line indicates the mean for the group.

To determine the antiviral activity of luminal drug, which represents drug released from the IVR that is never taken up by or transits out of the tissue and cells, TZM-bl cells were challenged with HIV-1_Ba-L_ in the presence of two different dilutions (1:10 and 1:100) of the CVL samples. Consistent with the concentration of drug recovered (Table 2), which at both CVL dilutions exceeds the *in vitro* IC_90_ of IQP-0528 in TZM-bl cells of 2.7 ng/ml (not shown), cells challenged with virus in the presence of CVL were significantly protected from infection (Figure 3a and b). There was a small drop in activity in 1:100 dilution CVL samples. In contrast, no antiviral activity was found in the baseline CVL or samples collected from animals exposed to placebo IVRs.

Subtype C HIV is one of the most prevalent in East and South Africa, India, China and Nepal [28]. To further examine the antiviral activity of drug released from the IVR, we expanded the studies with CVL and used a more stringent *ex vivo* model. TZM-bl cells were cultured in transwell™ inserts and exposed to diluted gamma-irradiated CVL applied to the basolateral side. Gamma irradiation was used to prevent microbial contamination and had no impact on drug activity (data not shown). The cells and CVL were incubated overnight to allow drug equilibration prior to apical challenge with two different amounts (10 or 100 TCID_50_) of CCR5-tropic HIV-1 subtype C (isolate 97USNG30). CVL samples collected from animals treated with the IQP-0528 IVR provided at least partial protection against 10 TCID_50_, with samples collected at day 7 from 2 of 4 animals and at day 14 from 3 of 4 animals, affording complete inhibition in this assay (Table 3). Notably, the three samples affording protection had the highest drug concentrations at day 14. When cells were challenged with 100 TCID_50_, complete inhibition was observed only for samples collected at day 14 from the two animals with higher CVL IQP-0528 levels (PMD2 and PTE2). No protection was observed with samples collected at baseline or from animals treated with placebo rings.

**Table 3:** Antiviral activity of cervicovaginal lavage (CVL) against primary HIV-1 subtype C (97USNG30) in dual chamber system

		**HIV-1 Subtype C (97USNG30)**					
**Animal**	**Day**	**10 TCID**_**50**_			**100 TCID**_**50**_		
**IQP 1 (PMD2)**	0	+	+	+	+	+	+
	7	-	-	-	+	-	-
	14	-	-	-	-	-	-
**IQP 2 (PKP1)**	0	+	+	+	+	+	+
	7	+	-	+	+	+	+
	14	-	-	+	+	+	+
**IQP 3 (PHC2)**	0	+	+	+	+	+	+
	7	-	-	-	+	+	+
	14	-	-	-	+	+	+
**IQP 4 (PTE2)**	0	+	+	+	+	+	+
	7	+	-	+	+	+	+
	14	-	-	-	-	-	-
**Placebo 1 (PZA2)**	0	+	+	+	+	+	+
	7	+	+	+	+	+	+
	14	+	+	+	+	+	+
**Placebo 2 (PUG2)**	0	+	+	+	+	+	+
	7	+	+	+	+	+	+
	14	+	+	+	+	+	+

TZM-bl cells were exposed to CVL obtained at the indicated times (Day 0 (baseline), Day 7 or Day 14) from macaques treated with the IQP-0528 or placebo intravaginal ring and challenged with HIV (10 or 100 TCID_50_) in triplicate. Culture wells with luciferase activity above or below background levels from CVL exposure alone are marked (+) or (-), respectively.

**Figure 4:**
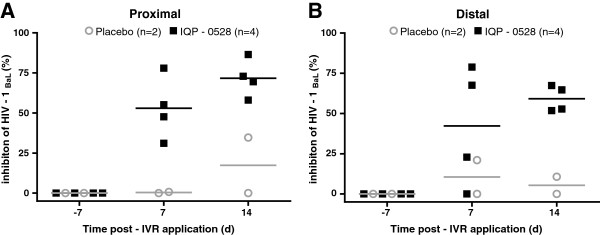
**Antiviral activity in NHP cervical tissue.** Proximal (cervical; **A**) and distal (introitus; **B**) biopsies collected at Day 0 (baseline) and 7 and 14 days post-insertion of IVR were minced and co-cultured with PHA-activated human PBMC (1x10^5^ cells/well) in triplicate wells of round-bottomed 96-well plates within 24 h of collection in the presence of culture medium used during shipment. Co-cultures were incubated at 37°C for 72 h prior to challenge with HIV-1_Ba-L_ (approximately 10^3^ TCID_50_). The inoculum was removed and co-cultured tissue and cells washed thrice with serum-free medium and incubated for 7–10 days at 37°C. Four animals were exposed to IVRs loaded with 10% (w/w) IQP-0528 (squares) and two animals received placebo IVRs (circles). HIV replication was assessed by measuring p24 levels by ELISA. Data are presented as the percent inhibition relative to control cells challenged in the absence of tissue co-culture and are means obtained from one experiment where each condition was tested in triplicate; the line indicates the mean for the group.

**Figure 5:**
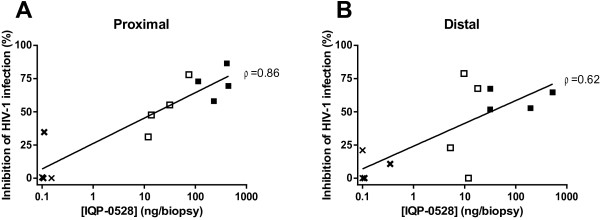
**PK/PD correlations.** Inhibition of HIV-1_Ba-L_ infection in co-cultures of proximal **(A)** and distal **(B)** biopsies was correlated with IQP-0528 levels in biopsy tissue. Crosses represent biopsies obtained from animals exposed to placebo IVRs. Open and closed squares represent biopsies collected from animals exposed to IQP-0528 IVRs at 7 and 14 days post-IVR insertion, respectively. The Spearman correlation coefficient (ρ) is indicated.

To evaluate IQP-0528 PD in biopsy tissue samples, biopsies collected from sites proximal and distal to the IVR insertion at baseline, 7 and 14 days post ring application were minced and co-cultured with PHA-activated human PBMC prior to challenge with HIV-1_Ba-L_. Co-culture with proximal biopsies yielded viral inhibition of 53.0% (day 7) and 71.7% (day 14), whereas distal biopsy co-cultures were relatively less protected, with 42.3% (day 7) and 59.2% (day 14) inhibition (Figure 4). The activity correlated with drug levels recovered (Table 2; Figure 5) with greater correlation observed with proximal (Spearman ρ = 0.86; p = 0.0006) than distal (Spearman ρ = 0.62; p = 0.03) biopsies, possibly reflecting less homogenous drug distribution as the distance to the site of release increases. One caveat of this analysis is that PK was measured using one set of biopsies and PD assessed in a different sample.

The combination of *in vitro* and *ex vivo* models evaluated in these studies provide a more comprehensive assessment of the potential activity of candidate microbicides under conditions that may better simulate clinical application of the drug. Specifically, the models allow for the assessment of key molecular properties that define the PD of the drug, including how fast and to what extent drugs traffic in and out of tissue and whether sufficient drug is retained at the site of its activity after a potential washout following exposure to SP. These models could also provide additional rationale for selecting drugs to be co-formulated for HIV prevention. Combinations that target different steps in the viral life cycle, act in different compartments (luminally, cell surface, or intracellularly) and, as indicated by the models here, exhibit complementary molecular properties that influence the cellular and tissue biodistribution of the drug may be optimal. By quantifying drug concentration and antiviral activity, combinations and target drug concentrations with the greatest potential for HIV prevention can be selected. For example, the combination of TDF and IQP-0528 could provide greater protection than achieved by either drug alone. TDF would protect resident cells even after a partial washout of the NNRTI by ejaculate because its active metabolite, TFV-DP, is retained intracellularly, whereas the tissue reservoirs of IQP-058 (or presumably, dapivirine) would provide sufficient drug to protect cells recruited into the genital tract.

There were also notable differences between the two entry inhibitors evaluated in these models. PIE12 retained substantial activity in both models, possibly reflecting its potency, membrane-localizing cholesterol group, and larger molecular weight compared to small molecules (~9 kDa) that likely slows diffusion into and out of tissues. In contrast, MVC, which was present prior to or during viral inoculation but was not re-added to the culture medium post infection, was readily washed out by SP (or medium) and failed to protect T cells in the co-culture model suggesting that, in the absence of sustained delivery, MVC may not have the PK properties needed to protect against infection. A recent study showed that the efficacy of MVC may be underestimated by measuring plasma viral loads because virus that is blocked from entering the cells may reenter the extracellular space [29]. However, it is unlikely that this property contributed to the findings of reduced activity in the models described here where the cells and tissue were washed.

There are several unique attributes to the co-culture model of PK/PD relationships. While direct challenge of biopsy tissue offers theoretical advantages, it has proven to be technically challenging. The co-culture model circumvents several of these problems. First, it overcomes the problem of reproducibility because of the variability in the number and activation status of the resident immune cells. Second, it can be applied to frozen tissue, whereas direct challenge should be performed with fresh tissue, as reproducibility infecting tissue that has previously been frozen has been poor compared to fresh tissue (Charlene Dezzutti; personal communication) and frozen tissue does not support HIV replication as well as fresh tissue [27]. Finally, in the case of NHP studies, the challenge can be performed with HIV, rather than SHIV or SIV because the NHP tissue is serving primarily as the source of drug, and not providing the immune target cells. Thus, co-cultures can be performed with human PBMCs (or a T cell line) as in Figure 2 and Table 1. The model assay can be easily expanded to test primary isolates, different clades, and founder viral populations. Challenges can also be performed with virus mixed with semen or seminal plasma.

## Conclusions

Drug combinations for topical prevention of HIV transmission have been proposed for more than a decade [30-32]. Potential advantages include the decreased likelihood of selecting for resistant viral variants, protection against resistant variants circulating in the community, and greater efficacy resulting from the synergy of targeting different stages of the HIV life cycle. The data presented here indicate that in addition to these attributes, combining drugs with complementary physical properties and biodistribution profiles could be key to protecting cells resident in the genital tract at the time of sexual exposure and those subsequently recruited into the mucosa. Importantly, these data highlight the importance of model assay systems to test candidate ARVs and ARV combinations.

## Methods

### Cells and viruses

Jurkat-Tat-CCR5 (JT-CCR5) cells were provided by Quentin Sattentau (Sir William Dunn School of Pathology, University of Oxford, Oxford, United Kingdom) and TZM-bl cells were obtained from the NIH AIDS Reagent Program and were both cultured as previously described [33]. PBMCs were obtained by density gradient centrifugation of healthy donor blood (venipuncture) diluted in Hanks balanced saline solution (HBSS) using Ficoll-paque™ Plus (GE Healthcare, Uppsala, Sweden) and activated by 3 day culture (5×10^5^cells/mL) in RPMI-1640 medium containing L-glutamine, 10% fetal bovine serum (FBS), 2 mM L-glutamine, 100 U/mL penicillin (herein referred to as complete medium), supplemented with 5 μg/mL Phytohemagglutinin (PHA) and subsequently cultured in complete medium supplemented with 50 U/mL interleukin (IL)-2 at 37°C in a humidified atmosphere containing 5% CO_2_. The laboratory-adapted HIV-1_Ba-L_ (CCR5-utilizing strain) was grown as previously described [33]. The Clade C strain 97USNG30 (NIH AIDS Research and Reference Reagent Program) was grown in human PBMCs. HIV stocks were stored in liquid nitrogen or at -70°C.

### Drugs and reagents

Tenofovir disoproxil fumarate (TDF) was provided by Gilead (Foster City, CA). The pyrimidinedione IQP-0528 was provided by ImQuest BioSciences Inc. and compounded at 10 wt% in Tecoflex® EG-85A polyurethane (PU) with a Haake-MiniLab extruder at 150°C (Thermo Scientific) and pelletized using a variable-speed pelletizer (Randcastle Extrusion). The pelletized extrudate was injection molded into IVRs (25 mm outer diameter and 5 mm cross-section) using a Babyplast 6/10P micro-injection molding system (ALBA Enterprises) with injection molding temperatures of 167-173°C. IVRs had an average mass of 1.27 g ± 5 mg (mean ± SD, N = 10). Dapivirine (DPV) and Maraviroc (MVC) were provided by the International Partnership for Microbicides (IPM) and the cholesterol-PIE12 trimer (PIE12) was synthesized at the University of Utah [20]. Human semen was purchased from Lee Biosolutions, Inc. (St Louis, MO). Semen was clarified by centrifugation at 500 g for 10 min and the supernatant (seminal plasma) divided into aliquots and stored at -80°C.

### Toxicity assessment

JT-CCR5 cells were exposed to the highest concentrations of the panel of drugs for 24 h in 96-well round-bottomed plates (2×10^5^ cells/well). Plates were centrifuged at 500 *g* for 5 min and supernatants were discarded prior to each of three washing steps with 200 μL of medium. Cell viability was assessed using the CellTiter 96 cell proliferation assay (MTS; Promega).

### Intracellular drug retention

JT-CCR5 T cells in 24-well plates (1×10^6^ cells/well) were exposed to varying concentrations of drugs diluted in complete medium for 24 h. The cells were then washed with 10 mL of medium, medium containing 10% human seminal plasma (SP), or not washed and then washed cells were resuspended in complete medium prior to transfer to round-bottomed 96-well plates and exposed to 10^3^ TCID_50_ (50% tissue culture infective dose) HIV-1_Ba-L_ for 2 h. Plates were centrifuged at 500 *g* for 5 min and supernatants were removed prior to each of 3 washing steps with 200 μL of medium. Cells were subsequently cultured in 200 μL complete medium and HIV replication was assessed 6 d post-infection by measuring p24 antigen by ELISA [34].

### Co-culture model for assessment of drug PD

Cervical tissue was collected from pre-menopausal women undergoing therapeutic hysterectomies at the Albert Einstein–Montefiore Weiler Hospital (Bronx, NY, USA) under an IRB exempt protocol. Ectocervical tissue was cut into explants of approximately 3×3×3 mm [33,35] prior to 24 h exposure to panel of unformulated drugs in 24-well plates. Extracellular free drug was removed by immersing explants in phosphate buffered saline (PBS) containing Ca^2+^ before mincing explants into fragments smaller than 1 mm, which were resuspended in 200 μL complete medium and co-cultured with JT-CCR5 cells (1×10^5^ cells/well) in triplicate wells of round-bottomed 96-well plates. Co-cultures were incubated at 37°C for 72 h to allow released drug to be transferred from the tissue to T cells prior to challenge with HIV-1_Ba-L_ (approximately 10^3^ TCID_50_). The inoculum was removed and co-cultured tissue and cells washed thrice with medium. HIV replication was assessed by measuring p24 levels by ELISA (Figure 2a).

### Non-human primate (NHP) studies

A 14-day IQP-0528 IVR biodistribution study using pigtail macaque monkeys was performed following protocols approved by the CDC Institutional Animal Care and Use Committee according to the Guide for the Care and Use of Laboratory Animals as previously described [36]. Briefly, animals were anesthetized with ketamine (3-10 mg/kg) prior to all collection procedures. A total of six macaques were used with two animals receiving placebo IVR and four animals receiving IQP-0528 (10% w/w) IVR. A pediatric speculum and forceps were used to insert IVRs in the posterior vagina proximal to the ectocervix on day 0 and left in place for 14 days. Tissue samples were collected with biopsy forceps (Miltex Townsend # 30–1445) near the vaginal introitus (distal to the ring) and the ectocervix (proximal to the ring) and shipped at 4°C to the Herold laboratory overnight. CVL samples were obtained by washing the vaginal vault with 5 mL normal saline; CVL samples were divided into aliquots, shipped and stored at -80°C.

### Measurement of IQP-0528 levels

IQP-0528 in biological samples was extracted and analyzed using LC-MS/MS at the Center for Human Toxicology (Utah). The lower limit of quantification (LLOQ) for this assay was 1 ng/mL of matrix. To convert weight/weight concentrations of IQP-0528 (nanograms IQP-0528 per milligram of vaginal fluid or tissue) to molarity (μM), vaginal fluid and tissue densities of 1.0 g/mL were assumed.

### Antiviral activity of CVL

TZM-bl cells were plated at 3x10^4^/well and allowed to adhere overnight before exposure to 10^3^ TCID_50_ HIV-1_Ba-L_ in the presence of CVL diluted 1:10 and 1:100 in complete DMEM in triplicate wells. Virus and drugs were left in culture for 48 h at 37°C and then removed by washing once with 200 μl PBS. Cells were lysed in 100 μl luciferase cell culture lysis reagent (Promega) and stored at -80°C until assessed for luciferase activity using luciferase assay buffer (Promega). Alternatively, TZM-bl cells were plated at 5x10^3^/well in polyester transwell™ inserts with 1.0 μm pore size (Corning) and exposed basolaterally to CVL diluted 1:20 in complete DMEM overnight before apical challenge with the CCR5-tropic subtype C HIV isolate 97USNG30 (0, 10 and 100 TCID_50_). Wells with luciferase activity above background average + 2 standard deviations (cells exposed to CVL but no virus) were considered positive. To avoid contamination, frozen CVL was Gamma-irradiated on dry ice (1KGy) prior to use.

### Assessment of NHP samples in co-culture model

Proximal and distal biopsies were collected and placed in 200 μL of complete medium and shipped to the Herold laboratory at 4°C. Biopsies were minced without washing and co-cultured with activated human PBMC in the presence of culture medium used during transport. Challenge and assessment of HIV replication were performed as described for human cervical explant tissue (Figure 2a).

### Statistical analyses

Analyses were performed using GraphPad Prism (GraphPad Software, Inc.). Differences between treatment conditions were compared by Kruskal-Wallis or unpaired t-test.

## Abbreviations

ARVs: Antiretrovirals; CVL: Cerviocovaginal lavage; DPV: Dapivirine; IVR: Intravaginal ring; JT-CCR5: Jurkat-Tat-CCR5 cells; MTS: 3-(4,5-dimethylthiazol-2-yl)-5-(3-carboxymethoxyphenyl)-2-(4-sulfophenyl)-2H-tetrazolium; MVC: Maraviroc; NHPs: Non-human primates; NNRTIs: Non-nucleoside reverse transcriptase inhibitors; PD: Pharmacodynamics; PK: Pharmacokinetics; PIE12: D-peptide chol-PIE-12 trimer; PrEP: Pre-exposure prophylaxis; RTIs: Reverse transcriptase inhibitors; SP: Seminal plasma; STI: Sexually transmitted infections; TDF: Tenofovir disoproxil fumarate; TFV: Tenofovir; TFV-DP: TFV-diphosphate.

## Competing interests

MSK is a consultant and equity holder in Navigen, which is commercializing D-peptide inhibitors of HIV entry. RWB Jr. is a shareholder and co-founder of ImQuest BioSciences, which is developing IQP-0528 as a topical microbicide. The remaining authors declare that they have no competing interests.

## Authors’ contributions

PMMM carried out all *in vitro* and *ex vivo* experiments apart from HIV subtype C challenges, participated in the conception and design of the study, analyzed data and wrote the manuscript. PS carried out animal experiments. TJJ and RR formulated IQP-0528 for intravaginal ring delivery. TE conducted HIV subtype C challenges and helped to draft the manuscript. MSK, KWB and RWB Jr. provided reagents and participated in the writing of the manuscript. JMS, PFK and BCH. conceived of the study, participated in its design and coordination and wrote the manuscript. All authors read and approved the final manuscript.
